# The interaction of post-activation potentiation and fatigue on skeletal muscle twitch torque and displacement

**DOI:** 10.3389/fphys.2024.1527523

**Published:** 2025-01-30

**Authors:** Georg Langen, Frank Warschun, Olaf Ueberschär, Michael Behringer

**Affiliations:** ^1^ Department of Sports Sciences, Goethe University, Frankfurt, Germany; ^2^ Department of Strength, Power and Technical Sports, Institute for Applied Training Science, Leipzig, Germany; ^3^ Department of Biomechanics and Sport Technology, Institute for Applied Training Science, Leipzig, Germany; ^4^ Department of Engineering and Industrial Design, Magdeburg-Stendal University of Applied Sciences, Magdeburg, Germany

**Keywords:** skeletal muscle, contractile properties, fatigue, post-activation potentiation, tensiomyography

## Abstract

**Introduction:**

Tensiomyography (TMG) assesses skeletal muscle contractile properties based on the electrically stimulated radial muscle displacement. As the relationship between twitch displacement and associated torque is poorly understood, it is unclear how it is affected by post-activation potentiation and muscle fatigue. This study investigated how the interaction of potentiation and fatigue affects the rectus femoris (RF) twitch displacement and associated torque.

**Materials and methods:**

Sixteen resistance-trained men (n = 8) and women (n = 8) performed two sets of five and five sets of ten seated maximum voluntary isometric knee extensions to induce potentiation and fatigue. Twitch displacement and torque were measured at baseline before the first set, after each set, and every 2 min for 15 min after the last set.

**Results:**

The exercise effectively induced potentiation and fatigue as peak twitch torque increased by 44.1% after the first set, decreased by 32.9% after the last set and remained decreased by 26.4% after 15 min. Twitch displacement was considerably less affected by the exercise. Consequently, TMG parameters could not accurately detect potentiated or fatigued participants as indicated by the peak twitch torque.

**Discussion:**

The TMG parameters’ insufficient diagnostic accuracy likely resulted from a reduced signal-to-noise ratio at 90° knee flexion and the associated longer muscle length of the RF, compared to more extended knee angles commonly employed in TMG studies. These results highlight an important methodological consideration as the joint angle, i.e. muscle length, appears to influence the TMG parameters’ ability to detect exercise-induced changes in contractile properties.

## 1 Introduction

Tensiomyography (TMG) is a non-invasive method to assess the contractile properties of skeletal muscles. TMG measures the radial displacement of a selected muscle during a single twitch contraction in response to a short electric stimulus (1 ms) (García-García et al., 2019). It is well known that any contractile activity performed prior to a twitch contraction affects the torque produced during the twitch (Hodgson et al., 2005). The effect on the twitch torque depends on the characteristics of the preceding activities, e.g., contraction mode, intensity, duration, volume, and resting time (Skurvydas et al., 2016; Tillin and Bishop, 2009). Previous studies have shown that a brief maximal voluntary contraction leads to a transient increase in the peak torque of a subsequent muscle twitch (Hamada et al., 2000; Pääsuke et al., 2007; Requena et al., 2008). This phenomenon is known as post-activation potentiation (PAP) (Blazevich and Babault, 2019). However, if the contractile activity is prolonged, e.g., by repeating maximum voluntary contractions, a reversible decrease in the twitch peak torque can be observed (Hamada et al., 2003; Chalchat et al., 2020), in terms of peripheral muscle fatigue (Allen et al., 2008). While potentiation and fatigue are two opposing phenomena, they can coexist within the same muscle (Rassier and Macintosh, 2000; Fowles and Green, 2003), and, consequently, the contractile properties assessed by a twitch contraction reflect the net balance between potentiation and fatigue at the time of measurement (Tillin and Bishop, 2009).

Compared to the twitch torque, only little is known about the effect of the post-activation-potentiation and muscle fatigue on the electrically induced radial displacement of the muscle belly, assessed via TMG. Therefore, Macgregor and colleagues suggested integrating TMG-derived parameters with other markers of muscle function under different physiological conditions (Macgregor et al., 2018). However, only very few studies have investigated the effect of potentiation on muscle displacement assesses via TMG (Herring et al., 2021; Paula Simola R. A. et al., 2015; Abazovic et al., 2022). In contrast, many studies have previously used TMG to assess muscle fatigue (Martín-San Agustín et al., 2020; García-García et al., 2020; Muñoz-López et al., 2020; Gasparini et al., 2012). However, in a recent systematic review with meta-analysis, Lohr and colleagues concluded that only limited evidence for the diagnostic accuracy of TMG concerning muscle fatigue exists (Lohr et al., 2019).

Since the effects of potentiation and fatigue on the twitch torque have been extensively investigated, comparing displacement and torque parameters derived from the same twitch offers a reasonable approach to help understand the relationship between muscle displacement and function. However, only four studies have compared the twitch displacement to the twitch torque during the same single twitch (Simunic et al., 2010; Koren et al., 2015; Abazovic et al., 2022; Kalc et al., 2023) or a double twitch (Kalc et al., 2023). While two of these studies, i.e. (Simunic et al., 2010) and (Koren et al., 2015), compared the twitch torque and displacement at rest, Abazovic and colleagues investigated their relationship following 5 × 5 s maximum voluntary isometric contractions (MVIC) to induce PAP (Abazovic et al., 2022). In this study, following the MVICs, both the displacement and torque peak amplitudes of the vastus lateralis (VL) and medialis (VM) muscles significantly increased for several minutes (Abazovic et al., 2022). However, while the displacement contraction time was significantly shorter after the MIVICs, the torque Tc was significantly increased for the VM and remained unaffected for the VL (Abazovic et al., 2022). In a recent study, Kalc and colleagues investigated the diagnostic accuracy of TMG parameters to detect muscle fatigue following a sustained submaximal MVIC and a 30 s all-out cycling test (Kalc et al., 2023). In this study, the VL twitch displacement was compared to the double twitch and MVIC torque from the whole quadriceps group (Kalc et al., 2023). The results of this study showed that TMG parameters could correctly classify 50%–76% as fatigued or not fatigued, as indicated by the double twitch torque response and 37%–63%, as assessed by the MVIC torque output (Kalc et al., 2023).

However, it remains unclear how muscle displacement is affected by the interaction of potentiation and fatigue and how it relates to the torque generated during the same twitch. Therefore, this study aimed to investigate how muscle displacement assessed via TMG is affected by the interaction of potentiation and fatigue in direct comparison to the torque produced during the same twitch. We hypothesized that throughout 60 repeated maximum voluntary isometric contractions, the peak amplitude of both the twitch torque and displacement would initially increase in the sense of potentiation and then gradually decrease as a result of muscle fatigue. We also hypothesize that the tensiomyographically determined twitch displacement parameters can accurately confirm the presence or absence of a potentiation or fatigue condition determined by the peak twitch torque.

## 2 Materials and methods

### 2.1 Study design, ethical approval, and pre-registration

This study employed a cross-sectional, single-group, within-participants repeated measures design. The design of this study followed the guidelines for sex and gender equity in research (SAGER) (Heidari et al., 2016). Further, this study complied with the principles established in the Declaration of Helsinki (World Medical Association, 2013). Before the recruitment and data collection, this study was approved by the local Ethics Committee (reference number: ER_2023.31.03_6) and pre-registered at the Open Science Framework platform. The pre-registration, data management plan, complete data and analysis code associated to this study are openly available at https://doi.org/10.17605/OSF.IO/SA8V5. All included participants gave their written informed consent to participate in this study without receiving any compensation.

### 2.2 Experimental approach

This study was conducted from June to August 2023 at the Institute for Applied Training Science in Leipzig, Germany. Participants were recruited by word of mouth and distribution of information material, such as flyers or emails, at the Institute of Applied Training Science in Leipzig, the University of Leipzig and local training facilities, e.g., commercial and public gyms.

All participants attended the lab on two occasions. The first appointment served as a familiarization session, during which we screened the subjects for eligibility, collected their anthropometric data and introduced them to all procedures and equipment.

**FIGURE 1:**
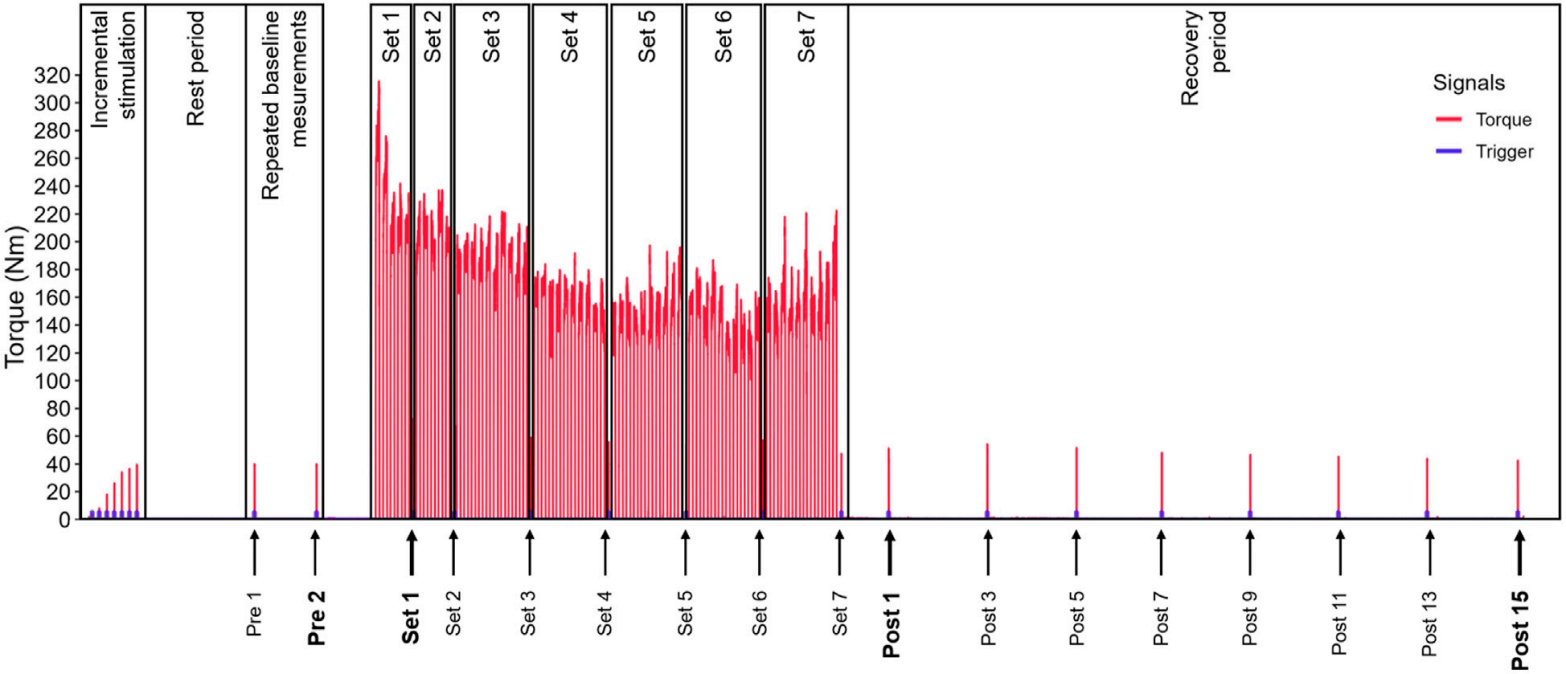
Schematic illustration of the chronological measurement sequence and exercise protocol including a representative torque trace.

On the second appointment, the participants performed a standardized warm-up protocol and then sat on the dynamometer chair for a rest period of 15 min. During this rest period, we collected information on the participants’ physical activity level and resistance training status. Next, we determined the RFs maximum twitch displacement and simultaneous twitch torque via stepwise incremental electrical stimulation. After another rest period of 3 min, we recorded two single twitch responses interspersed by 1 min of rest at the individual maximum stimulation intensity. The displacement sensor was removed between these repeated measurements to match the subsequent measurements during the exercise protocol. These two twitch responses were used to assess the reproducibility of the twitch torque and displacement parameters (repeated baseline measurements, see Figure 1). The twitch parameters’ reproducibility was assessed to ensure we would be able to differentiate real change from random variation within the actual test setting.

The participants then performed the exercise protocol, which consisted of 60 maximum voluntary isometric knee extensions, split into two sets of five (Set 1 and Set 2, see Figure 1) and five sets of ten contractions (Set 3 to Set 7, see Figure 1). A single twitch response was recorded and later used for analysis during the rest period after each set, immediately after the last set, 1 minute after the last set and then every 2 min until 15 min after the last set (see Figure 1).

All participants were asked to refrain from strenuous physical exercise and alcohol consumption within 48 h before the second appointment to ensure they would be well-rested (Barnes, 2014). Also, participants were asked to refrain from consuming any caffeine on the day of the second appointment before the measurements to prevent any potential confounding (Domaszewski et al., 2021).

### 2.3 Participants

We recruited 16 subjects (eight women and eight men) who met the following inclusion criteria: healthy, aged 18–40 years, experienced in resistance training, including lower body exercises, for at least 6 months with a minimum of two training sessions per week. Participants were excluded from this study based on the following criteria: pregnancy, history of neuromuscular, musculoskeletal or cardiovascular disorders, pain or injury in the lower limbs during the last 6 months, nontolerance or any contraindication to electrical stimulation using self-adhesive electrodes, and wearing an implantable medical device. Table 1 shows the anthropometric characteristics and strength training experience of the participants.

**TABLE 1:** Anthropometric characteristics and strength training status of the participants.

	All (n = 16)	Women (n = 8)	Men (n = 8)
Age (years)	26.94 ± 4.01	25.62 ± 3.20	28.25 ± 4.50
Height (cm)	175.70 ± 8.07	170.14 ± 7.39	181.26 ± 3.79
Weight (kg)	73.04 ± 10.79	64.84 ± 8.51	81.24 ± 4.85
Current training period (months)	13.44 ± 8.59	15.62 ± 10.60	11.25 ± 5.90
Session per week	2.94 ± 0.85	3.12 ± 0.99	2.75 ± 0.71
Time per session (minutes)	75.94 ± 22.23	67.50 ± 22.68	84.38 ± 19.54
Detraining period (months)	3.53 ± 4.79	2.38 ± 3.06	4.69 ± 6.07
Previous training period (months)	16.56 ± 29.73	18.62 ± 40.35	14.50 ± 16.00

To provide a comprehensive description of our sample, the participants’ strength training status was determined according to the model proposed by Santos Junior et al. (2021). Accordingly, one male participant was classified as beginner, 12 participants (five females, seven males) were classified as intermediate and three females were classified as advanced. Further, we determined the participants’ physical activity level via the short form of the International Physical Activity Questionnaire (IPAQ-SF) (Craig et al., 2003), which is presented in Table 2. According to the IPAQ-SF, the physical activity level of four participants (two females and two males) was classified as moderate, and the level of twelve participants (six females and six males) was classified as high.

**TABLE 2:** Physical activity level of the participants.

		All (n = 16)	Women (n = 8)	Men (n = 8)
Time spent on vigorous PA (min/week)	Median (q1, q3)	25.0 (24.0, 29.2)	24.5 (24.0, 25.5)	27.5 (24.0, 31.0)
Time spent on moderate PA (min/week)		75.3 (63.3, 82.3)	62.8 (59.8, 66.3)	81.4 (77.6, 83.9)
Time spent walking (min/week)		240.0 (180.0, 457.5)	210.0 (135.0, 270.0)	405.0 (240.0, 480.0)
Time spent sitting (min/week)		210.0 (90.0, 420.0)	210.0 (140.0, 420.0)	285.0 (90.0, 375.0)
Energy expended on PA (MET-min/day)		130.0 (72.5, 180.0)	160.0 (45.0, 191.2)	120.0 (110.0, 180.0)

PA: physical activity; q1: 25th percentile; q3: 75th percentile.

### 2.4 Sample size justification

We performed an *a priori* sample size calculation for a repeated-measures within-participants analysis of variance (ANOVA) with one group and 23 measurements using g*power v 3.1.9.7. We used an alpha error probability of 0.01, a power of 0.9 and a non-sphericity correction of 0.1 for the calculation. Further, we assumed an effect size of f = 1.49 for the change in the peak twitch torque, which we calculated from the data previously published by Siracusa and colleagues (2019). A sample size of seven subjects was required based on these input parameters. However, we partially adopted the test protocol from Siracusa et al. (2019) with slight modifications, including an additional 10 s rest after the first five MVICs, and we also included women in our sample. Therefore, we decided to include 16 subjects in our study to account for a possibly smaller effect. When assuming a sample size of 16 subjects and the same input parameter as above, the required effect size would be f = 0.76 to find a significant change in the peak twitch torque over time. Likewise, the required effect size for a gender-differentiated group of 8 subjects would be f = 1.20 to find a significant change, assuming the same input parameters described above.

### 2.5 Familiarization

During the familiarization session, the participants were introduced to the standardized warm-up protocol. The protocol consisted of low-intensity (75 W) cycling on a cycle ergometer (ergo_bike 8008 TRS, Daum Electrocnic, Fürth, Germany) for 5 minutes at a cadence between 80 and 90 rpm (Ditroilo et al., 2013). Afterwards, they sat on an IsoMed 2000 dynamometer (D. and R. Ferstl GmbH, Hernau, Germany) with a hip angle of 95° flexion (0° = hip extended according to neutral zero method) and a knee angle of 90° flexion (0° = knee fully extended according to neutral zero method). A foam rubber padded stiff adapter attached to the lever arm of the dynamometer was positioned 5 cm superior to the lateral malleolus. The lower leg was fixed to the adapter using a clamping strap. The dynamometers’ axis of rotation was visually aligned to the lateral epicondyle of the knee. Further, the participants were fixed firmly to the dynamometer chair using belts across the chest and pelvis. During all measurements, the participants were instructed to cross their arms in front of their chest. The individually adjusted dynamometer settings were recorded for the second appointment. On the second appointment, these settings were double-checked and adapted if necessary.

Once the individual position on the dynamometer was set, we familiarized the participants with the measurement procedures. These included measuring the RFs electrically stimulated maximum radial displacement. For these measurements, we used a TMG-S1 electrical stimulator (TMG-BMC d. o.o., Ljubljana, Slovenia), a GD30 displacement sensor (Panoptik d. o.o., Ljubljana, Slovenia) and two squared self-adhesive electrodes (50 × 50 mm, Axion GmbH, Leonberg, Germany). All measurements were performed by the same investigator (GL), who is experienced in performing TMG measurements for more than 4 years. We recorded the signal of the displacement sensor using the TMG Software v3.6 (TMG-BMC d. o.o., Ljubljana, Slovenia). Before any electrical stimulation was applied, we cleaned the skin in the measurement area with an electrode contact spray (Axion GmbH, Leonberg, Germany) and let it dry before positioning the sensor and the electrodes. Then, we defined the position of the sensor as follows: First, we determined the midpoint on a straight line between the superior border of the patella and the anterior superior iliac spine (Perotto et al., 2011). Second, within the area of the point determined during step one, we determined the thickest part of the muscle belly via inspection and palpation during a voluntary contraction of the knee extensors. Third, if necessary, we adjusted the position of the sensor at the beginning of the incremental electrical stimulation procedure to avoid any coactivation as indicated by a second peak in the displacement curve (Lohr et al., 2018; Macgregor et al., 2016). Finally, the position of the sensor was marked on the skin using a dermatological pen to ensure consistent positioning in subsequent measurements.

To apply the electrical stimulation, we positioned two electrodes on the skin above the rectus femoris muscle belly at a distance of 7 cm (Wilson et al., 2019a) between the facing edges of the electrodes and 3.5 cm to the position of the sensor, respectively. The electric stimulation consisted of single, monophasic, square wave stimuli, each of 1 ms duration. To familiarize the participants with the electrical stimulation procedure, we applied an incremental stimulation protocol, starting at 30 mA and increasing by 10 mA every 10 s until the individual maximum displacement of the RF or the maximum output of the stimulator was reached (Langen et al., 2023).

In addition to the radial displacement, we determined the simultaneously produced external torque during each stimulated twitch response. To this end, we recorded the analogue voltage signal of the IsoMed 2000 via a BNC-connected data acquisition device (DT9800, Measurement Computing Corporation, Norton, MA, United States) and the corresponding data acquisition software (QuickDAQ v.3.7.0.46, Measurement Computing Corporation, Norton, MA, United States). Using the same device and software, we also recorded an analogue trigger signal delivered by the BNC-connected TMG stimulator with every electric stimulus, which we used to synchronize the twitch displacement and torque responses.

To familiarize participants with the exercise protocol, they performed two sets of five maximum voluntary isometric knee extensions for 5 s each, with rest periods of 5 s between contractions and 10 s between sets. After both sets, we recorded a single twitch response at the previously determined individual maximum stimulation intensity.

All measurements were performed on the dominant leg, i.e., the leg subjects reportedly would use to shoot a ball at a target (van Melick et al., 2017).

### 2.6 Exercise protocol

The exercise protocol consisted of two sets of five MVICs followed by five sets of ten MVICs with rest periods of 5 s between contractions and 10 s between sets. Our protocol was intended to initially induce potentiation and then fatigue towards completion. To ensure that both goals would be met, on one hand, we adopted the protocol from of Abazovic et al. (2022). Their study showed that five sets of five MVICs with 5 s rest lead to an increase in the twitch torque of the knee extensors. On the other hand, our protocol was adopted from a previous study by Siracusa et al. (2019) who showed that six sets of ten 5-second MVICs with 5 s rest could effectively induce muscle fatigue of the knee extensors. However, compared to the study by Siracusa et al. (2019), we added an additional rest after the first five MVICs in order to maintain the comparability of our results with those of Abazovic et al. (2022).

Participants were instructed to hold each of the 60 MVICs for 5 s and perform each contraction at maximum intensity. A digital interval timer provided acoustic feedback to control the duration of MVICs and rest periods. The participants were verbally encouraged and received visual feedback on their torque production throughout the exercise, as the combination of verbal encouragement and visual feedback has been shown to increase the force output during maximum voluntary contractions (Miller et al., 2021; Amagliani et al., 2010; Campenella et al., 2000). The visual feedback was provided via a screen placed approximately 1 m in front of the participant at eye level. The screen showed a real-time torque graph, with the torque on the *y*-axis and the time on the *x*-axis. During each contraction, the participants were instructed to move the torque trace on the screen as high as possible by extending their knee as hard as possible against the dynamometer’s lever arm. Once they could not move the torque trace any higher, they were instructed to try to keep it at that level until the end of each five-second MVIC.

### 2.7 Data analysis

For each participant, we recorded the analogue voltage signal from the dynamometer for the entire session, from the start of the incremental electrical stimulation until the last stimulated twitch response. We then used a daily determined calibration factor to convert the analogue voltage signal from the dynamometer to torque. For each voluntary contraction, we determined the MVIC as the highest window of a rolling median (window width = 501 data points) within the period during which the torque was above 20 Nm. Stimulated twitch torque responses were identified via the trigger signal recorded from the electrical stimulator. For each twitch torque response, the torque signal was digitally filtered using a 4^th^-order low-pass Butterworth filter and a cut-off frequency of 15 Hz. From the filtered signal, we determined the peak of the respective torque times series data for each twitch response (Pt).

Further, for each stimulated twitch response, we recorded the radial displacement of the RF via the TMG software, which automatically calculated the first peak of the radial displacement curve (Dm, mm), the delay time (Td, ms) and the contraction time (Tc, ms). Several authors previously pointed out that changes in Td and Tc should not be interpreted independently of changes in Dm as they are partially dependent on the magnitude of radial displacement (Macgregor et al., 2016; Paula Simola R. A. et al., 2015). In settings where changes in Dm are expected, the rate of displacement has been suggested as a useful additional parameter, reflecting changes in contraction speed independent of changes in Dm, as it combines spatial and temporal aspects of the (Langen et al., 2022; Macgregor et al., 2018; García-García et al., 2020). Thus, from the data provided by the TMG software, we calculated the mean rate of displacement from the electrical stimulus until 10% of Dm (mm/s) (García-Manso et al., 2011) as ${Vc}_{0-10\%}=0.1\times Dm/Td\times 1000$ , the mean rate of displacement from the electrical stimulus until 90% of Dm (mm/s) (Paula Simola R. A. et al., 2015) as ${Vc}_{0-90\%}=0.9\times Dm/\left(Td+Tc\right)\times 1000$ , the mean rate of displacement between 10% and 90% of Dm (mm/s) (García-Manso et al., 2011) as ${Vc}_{10-90\%}=0.8\times Dm/Tc\times 1000$ and the normalized rate of displacement (1/s) (Valenčič and Knez, 1997) as ${Vc}_{norm}=0.8/Tc\times 1000$ .

### 2.8 Statistics

We checked all data for extreme values via inspection of boxplots and the Tukey method. Accordingly, extreme values were defined as data points outside 3 times the respective interquartile range. Any extreme values were checked for errors and removed if the accounting error could not be corrected. If no error could be identified, the data were transformed to comply with the assumptions of subsequent statistical procedures. Further, we checked if the data followed an approximal normal distribution via Shapiro-Wilk’s test and inspection of QQ plots. The level of statistical significance was set to *p* ≤ 0.05.

To assess the reproducibility of baseline measurements (PRE 1 and PRE 2), we performed a paired-samples *t*-test to test for a statistically significant systematic bias (Atkinson and Nevill, 1998) and determined bias-corrected effect sizes along with 95% confidence intervals (CI) (Lakens, 2013). We defined thresholds of 0.2, 0.6, 1.2, 2.0 and 4.0 for small, moderate, large, very large or extremely large effects (Hopkins et al., 2009). The relative reliability of baseline measurements was assessed by intraclass correlation coefficients (ICC, single rating (k = 1), absolute agreement, two-way mixed effects model) with 95% CI (Koo and Li, 2016). ICC values of <0.5, between 0.5 and 0.75, between 0.75 and 0.9 or >0.9 were interpreted as poor, moderate, good or excellent relative reliability (Koo and Li, 2016). The absolute reliability was assessed by the absolute and relative Standard Error of Measurement (SEM and SEM%, respectively), calculated as $SEM=SD-\sqrt{\left(1-ICC\right)}$ (Atkinson and Nevill, 1998), with SD referring to the standard deviation of all scores of PRE 1 and PRE 2, and $SEM\%=\left(SEM/M\right)\times 100$ (Wagner et al., 2008), with M referring to the mean of all scores of PRE 1 and PRE 2. Further, we calculated the absolute and relative minimal detectable change (MDC and MDC%, respectively) as $MDC=SEM\times 1.96\times \sqrt{2}$ (Atkinson and Nevill, 1998; MDC%=MDC/M×100 (Wagner et al., 2008).

To test for an effect of repeated MVICs on the last MVIC of each set and stimulated twitch responses over time, we conducted a one-way within-subjects repeated-measures ANOVA. In the case of a statistically significant change over time, we performed Bonferroni-corrected paired t-tests between the first MVIC and the last MVIC of each set or, for twitch parameters, Pre 2 and the subsequent measurement time points, respectively, to identify statistically significant changes in MVIC torque or stimulated twitch responses from baseline. We also calculated bias-corrected effect sizes with 95% CIs for pairwise comparisons. Additionally, to assess the relative decline in MVIC torque throughout the exercise, we calculated the fatigue index (%) as $FI=\left(first\;MVIC-last\;MVIC\right)/first\;MVIC\times 100$ (Jeon and Griffin, 2018).

Further, we performed a Pearson product-moment correlation analyses to assess if percentage changes from baseline at Set1, Post1 and Post15 in the twitch displacement parameters were associated with percentage changes in the peak twitch torque. According to (Hopkins et al., 2009), we defined thresholds of 0.1, 0.3, 0.5, 0.7 and 0.9 for small, moderate, large, very large and extremely large correlation coefficients.

Lastly, we assessed the diagnostic accuracy of twitch displacement parameters for fatigue and potentiation in reference to the peak twitch torque. Therefore, we classified subjects as potentiated or non-potentiated at Set1 and as fatigued or non-fatigued at Post1 and Post15 based on the MDC of the respective variable. If the difference between baseline and Set1, Post1 or Post15 for a given variable and a given subject exceeded that variable’s MDC, the respective subject was classified as potentiated or fatigued. Specifically, an increase from baseline exceeding the MDC resulted in a classification as potentiated, and a decrease from baseline exceeding the MDC resulted in a classification as fatigued.

The assessment of diagnostic accuracy followed the steps as previously suggested (Dhamnetiya et al. 2022): First, based on the classification for Set1, Post1 and Post15, we constructed the respective 2 × 2 contingency table, where the reference test (peak twitch torque) was represented in columns and the index test (twitch displacement parameters) in rows. Second, from the contingency table, we calculated the sensitivity (S_n_) as $S_{n}=true\;positives/\left(true\;positives+false\;negatives\right)\times 100$ and the specificity (S_p_) as $S_{p}=true\;negatives/\left(false\;positives+true\;negatives\right)\times 100$ . Further, we calculated the positive predictive value (PPV) as $PPV=true\;positives/\left(true\;positives+false\;positives\right)\times 100$ and the negative predictive value (NPV) as $NPV=true\;negatives/\left(false\;negatives+true\;negatives\right)\times 100$ . We also calculated the diagnostic effectiveness (DE) as $DE=\left(S_{n}\times Prevalence\right)+S_{p}\times \left(1-Prevalence\right)$ and the Youden’s index (YI) as $YI=\left(S_{n}+S_{p}\right)-1$ . Lastly, we plotted a receiver operator characteristics (ROC) curve, i.e., a line representing S_n_ versus - S_p_. We also determined the area under the ROC curve (AUROC) as a summary measure of diagnostic accuracy. According to Šimundić (2009), if AUROC was <0.5, 0.5–0.6, 0.6–0.7, 0.7–0.8, 0.8–0.9 and 0.9–1.0, we interpreted the diagnostic accuracy as insufficient, bad, sufficient, good, very good and excellent, respectively.

## 3 Results

All 16 subjects tolerated the measurement procedures and the exercise protocol well and fully completed the study. Table 3 presents the descriptive statistics of all variables, mean differences between repeated baseline measurements (Pre 1 and Pre 2), and relative and absolute reliability measures.

**TABLE 3:** Twitch parameters at Pre 1 and Pre 2, mean differences between time points and measures of relative and absolute reliability.

Variable	Pre 1 (mean ± sd)	Pre 2 (mean ± sd)	∆ (95% CI)	N	Test statistic, *p*-value	Hedge’s g (95% CI)	ICC (95% CI)	SEM	SEM%	MDC	MDC%
Pt (Nm)	19.47 ± 8.49	19.38 ± 8.50	0.09 (−0.30–0.48)	16	t = 0.508, *p* = 0.62	0.01 (−0.03–−0.04)	1.00 (0.99–1.00)	0.50	2.56	1.38	7.09
Dm (mm)	3.06 ± 1.86	2.88 ± 1.73	0.17 (−0.10–0.45)	16	t = 1.364, *p* = 0.19	0.09 (0.08–0.20)	0.96 (0.88–0.98)	0.36	12.26	1.01	33.97
Td (ms)	20.45 ± 9.96	18.77 ± 4.21	1.68 (−2.13–5.50)	16	t = 0.941, *p* = 0.36	0.15 (−0.04–−0.04)	0.56 (0.12–0.82)	5.00	25.51	13.87	70.72
Tc (ms)	21.64 ± 2.41	21.73 ± 2.39	−0.09 (−0.72–0.54)	16	t = −0.313, *p* = 0.76	−0.04 (0.04–0.12)	0.88 (0.70–0.96)	0.80	3.69	2.22	10.24
Vc_0–10%_ (mm/s)	13.59 ± 7.81	12.84 ± 7.38	0.75 (−0.17–1.67)	16	t = 1.744, *p* = 0.10	0.09 (−0.07–−0.09)	0.97 (0.92–0.99)	1.27	9.63	3.53	26.71
Vc_0–90%_ (mm/s)	62.90 ± 36.49	61.37 ± 34.82	1.53 (−1.87–4.92)	16	t = 0.959, *p* = 0.35	0.04 (0.07–0.11)	0.98 (0.96–0.99)	4.42	7.12	12.26	19.73
Vc_10–90%_ (mm/s)	118.16 ± 69.84	117.26 ± 65.66	0.90 (−7.05–8.86)	16	t = 0.241, *p* = 0.81	0.01 (−0.45–−0.18)	0.98 (0.94–0.99)	10.08	8.56	27.95	23.74
Vc_norm_ (1/s)	44.58 ± 14.45	44.92 ± 11.16	−0.34 (−3.14–2.46)	16	t = −0.256, *p* = 0.80	−0.02 (0.25–0.14)	0.92 (0.79–0.97)	3.56	7.95	9.86	22.03

∆: Mean difference between Pre 1 and Pre 2; N: number of participants analyzed; ICC: Intraclass-correlation coefficient; SEM: standard error of measurement; MDC: minimal detectable change.

### 3.1 Reproducibility of baseline measurements

There were no statistically significant differences between Pre 1 and Pre 2 for any twitch parameters, with the respective effect sizes indicating mostly trivial and at most small differences (Table 3). The relative reliability was good to excellent for all twitch parameters except for Tc, which showed poor to good reliability. The absolute reliability was best for Pt, as shown by the lowest SEM% and MDC%. As for the twitch displacement parameters, SEM% and MDC% were the lowest for Tc and the highest for Dm and Td. Across Vc concepts, Vc_0–90%_ showed the best absolute reliability.

### 3.2 ANOVA

#### 3.2.1 MVIC torque

**FIGURE 2:**
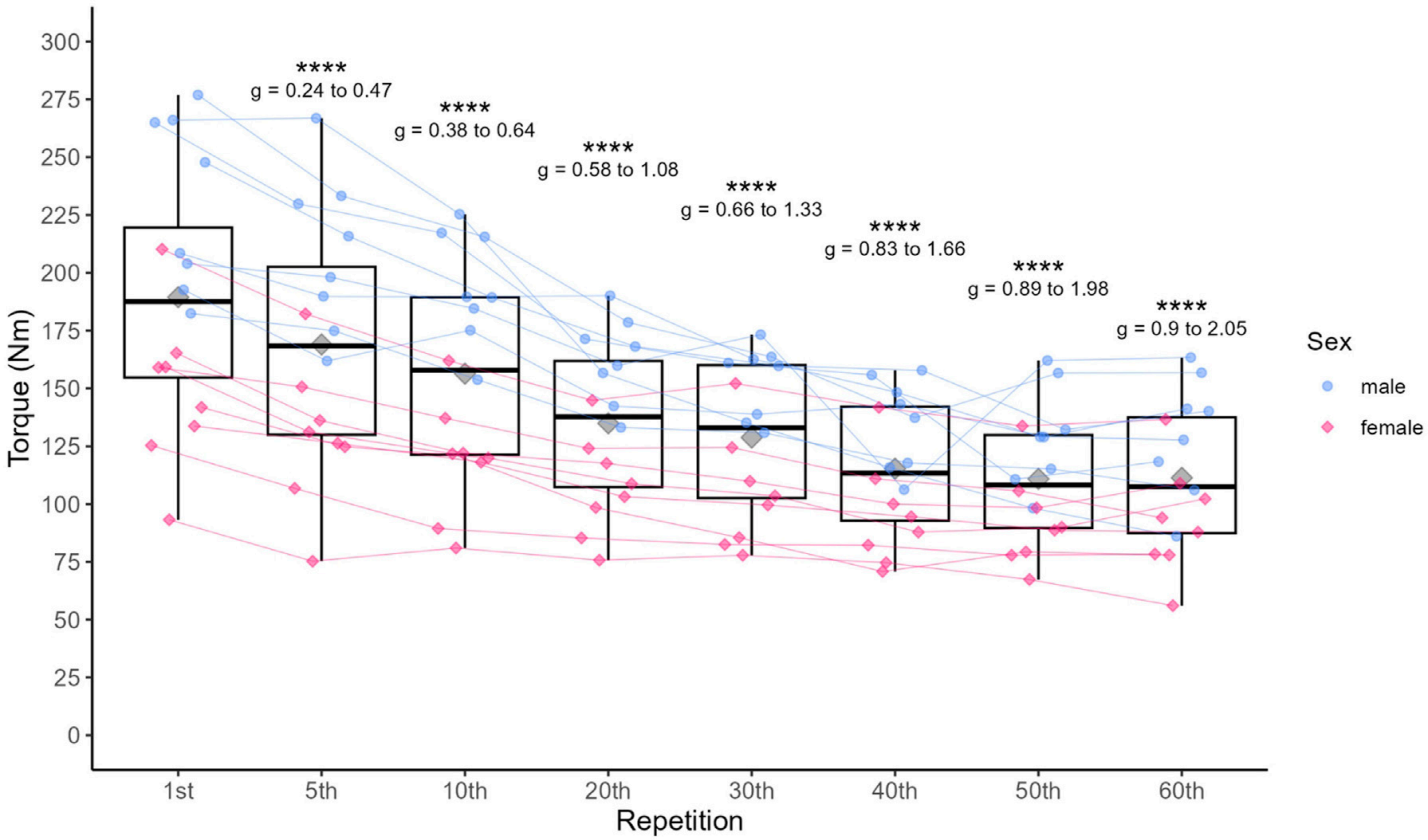
Individual data points for MVIC and statistically significant differences compared to the first repetition in the first set, including 95% confidence limits of the respective effect sizes. Diamonds represent the mean, thick horizontal lines the median, top and bottom end of the box correspond to the 75th and 25th percentiles, respectively. Whiskers extend to 1.5 times the distance between 25th and 75th percentile from the top and bottom end of the box, respectively. *p < 0.05, ** <0.01, ***p < 0.001, ****p < 0.0001.

MVIC torque decreased significantly (*p* < 0.001) over the course of the exercise (see Table 4) with increasing effect sizes from g (95% CI) = 0.36 (0.24–0.47) at the end of the first set to g (95% CI) = 1.47 (0.90–2.05) at the end of the last set, overall ranging from small to large effects (see Figure 2). The FI (mean ± sd) for MVIC torque was 39.8% ± 11.5%, ranging from 19.9% to 67.7%.

**TABLE 4:** ANOVA results for MVIC torque and twitch parameters.

Variable	N	DF_n_	DF_d_	F	p	η^2^
MVIC	16	1.62	24.29	38.16	<0.001	0.718
Pt	16	2.60	38.96	36.51	<0.001	0.709
Dm	16	2.52	37.85	6.32	0.002	0.296
Td	16	3.72	55.75	5.38	0.001	0.264
Tc	16	3.87	58.05	6.08	<0.001	0.288
Vc_0–10%_	16	2.40	35.97	9.12	<0.001	0.378
Vc_0–90%_	16	2.42	36.37	9.35	<0.001	0.384
Vc_10–90%_	16	2.49	37.42	9.00	<0.001	0.375
Vc_norm_	16	1.58	23.63	1.12	0.329	0.070

N: number of participants analyzed; DF: degrees of freedom; F: F-value; p: *p*-value; η^2^: partial eta squared.

#### 3.2.2 Stimulated twitch responses

In one participant, the sensor did not detect any radial displacement of the RF at time points Set 3 and Set 7. Therefore, the corresponding missing values for Dm, Td and Tc were estimated by linear interpolation (Noor et al., 2013). Specifically, the two missing values for the respective parameters and time points were replaced by the average of the previous and subsequent values of the same subject. The exercise protocol induced a statistically significant change in all twitch response parameters except for Vc_norm_ (Table 4). As there were several extreme values regarding Vc_norm_, we transformed the data accordingly and repeated the ANOVA, but found no statistically significant change. Thus, we did not perform *post hoc* pairwise comparisons for Vc_norm_.

**FIGURE 3:**
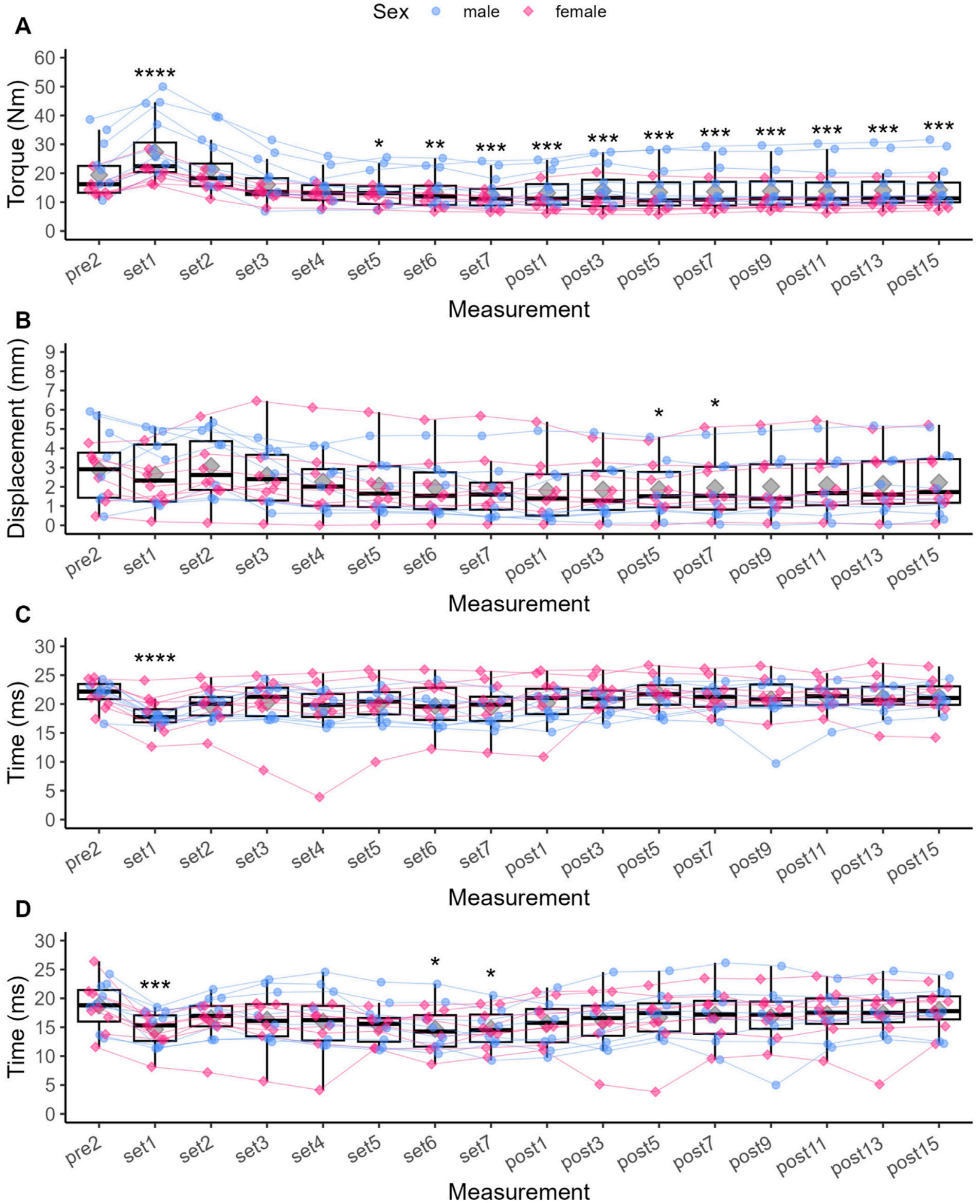
Individual data points for Pt **(A)**, Dm **(B)**, Td **(C)** and Tc **(D)**, respectively, and statistically significant differences compared to baseline (Pre 2). Diamonds represent the mean, thick horizontal lines the median, top and bottom end of the box correspond to the 75th and 25th percentiles, respectively. Whiskers extend to 1.5 times the distance between 25th and 75th percentile from the top and bottom end of the box, respectively. The ANOVA was not grouped by sex, but individual data points are shaped and coloured by sex following the SAGER guidelines. *p < 0.05, ** <0.01, ***p < 0.001, ****p < 0.0001.

**FIGURE 4:**
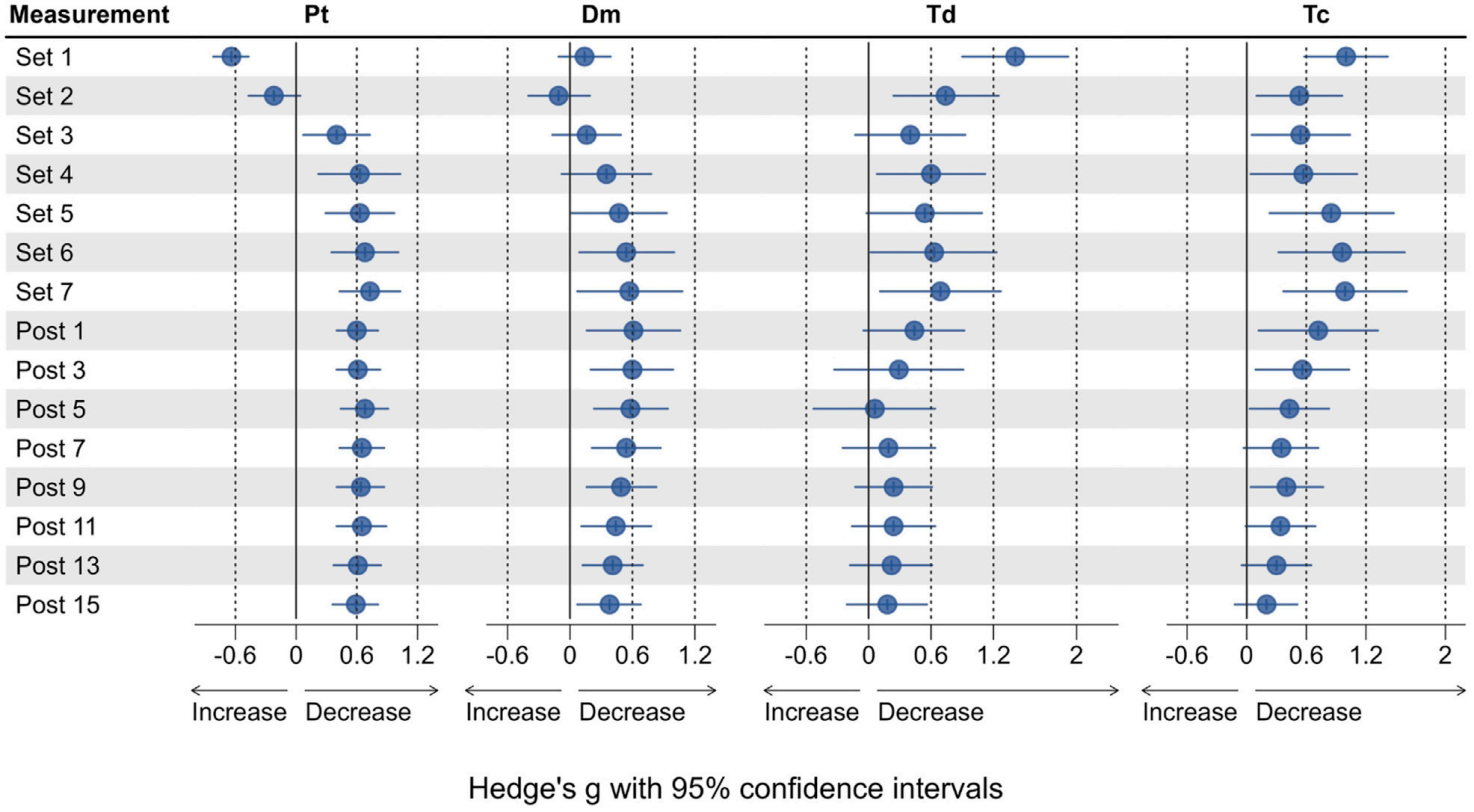
Effect sizes for Pt, Dm, Td and Tc, representing the change from baseline (Pre 2). Dots represent the estimates of the effect size, horizontal lines represent the 95% confidence intervals.

Compared to Pre 2, Pt was significantly increased by 44.1% after Set 1 (*p* < 0.001, see Figure 3), representing a small to moderate potentiation effect (see Figure 4). Pt then was significantly decreased from Set 5 to Post 15 (*p* < 0.05), reaching a maximum reduction of 32.9% at Set 7, representing a small to moderate fatigue effect. In contrast, Dm was decreased by 2.8% after Set 1 (*p* = 1.000) but increased by 26% after Set 2 (*p* = 1.000). At both time points, effect size confidence interval limits ranged from trivial to moderate effects (see Figure 4). From Set 3 to Post 15, Dm was decreased compared to Pre 2 with trivial to moderate effect size estimates. Statistically significant reductions in Dm could only be detected at Post 5 (39.1%, *p* = 0.034) and Post 7 (38.8%, *p* = 0.045). Td was significantly decreased by 17.1% after Set 1 (*p* < 0.001) with a moderate to very large effect size, and remained decreased until Post 15 with moderate to trivial effect size estimates (*p* > 0.05).

Compared to Pre 2, Tc was significantly decreased by 21% at Set 1 (*p* < 0.001) with a small to large effect size. Tc then remained decreased until Post 15, reaching statistical significance again at Set 6 (*p* = 0.031) and Set 7 (*p* = 0.018) with small to large effect sizes at both time points.

**FIGURE 5:**
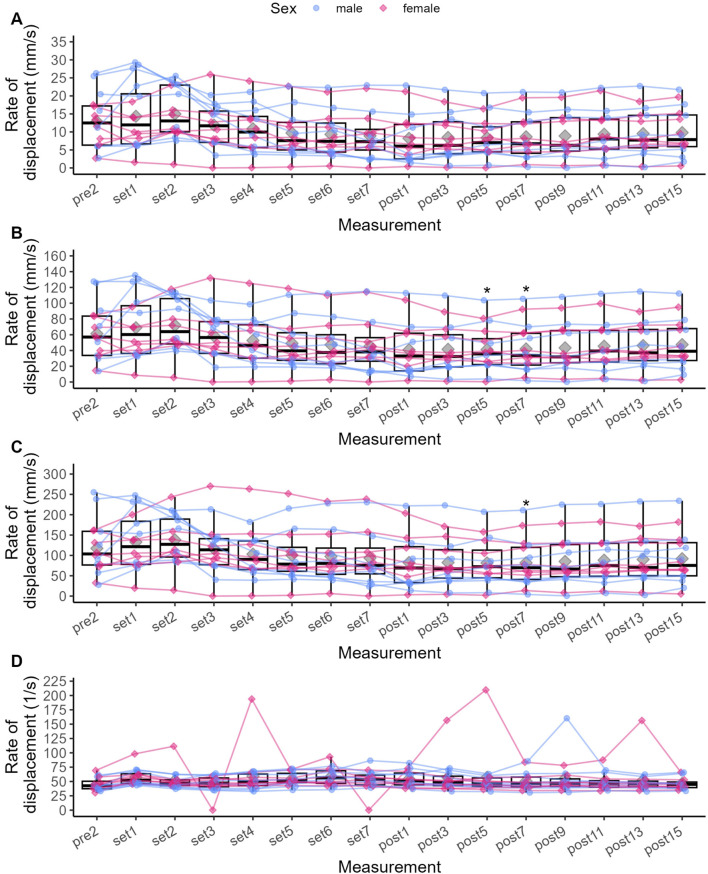
Individual data points for Vc_0–10%_ **(A)**, Vc_0–90%_ **(B)**, Vc_10–90%_ **(C)** and Vc_norm_ **(D)**, respectively, and statistically significant differences compared to baseline (Pre 2). Diamonds represent the mean, thick horizontal lines the median, top and bottom end of the box correspond to the 75th and 25th percentiles, respectively. Whiskers extend to 1.5 times the distance between 25th and 75th percentile from the top and bottom end of the box, respectively. The ANOVA was not grouped by sex, but individual data points are shaped and coloured by sex following the SAGER guidelines. *p < 0.05, ** <0.01, ***p < 0.001, ****p < 0.0001.

**FIGURE 6:**
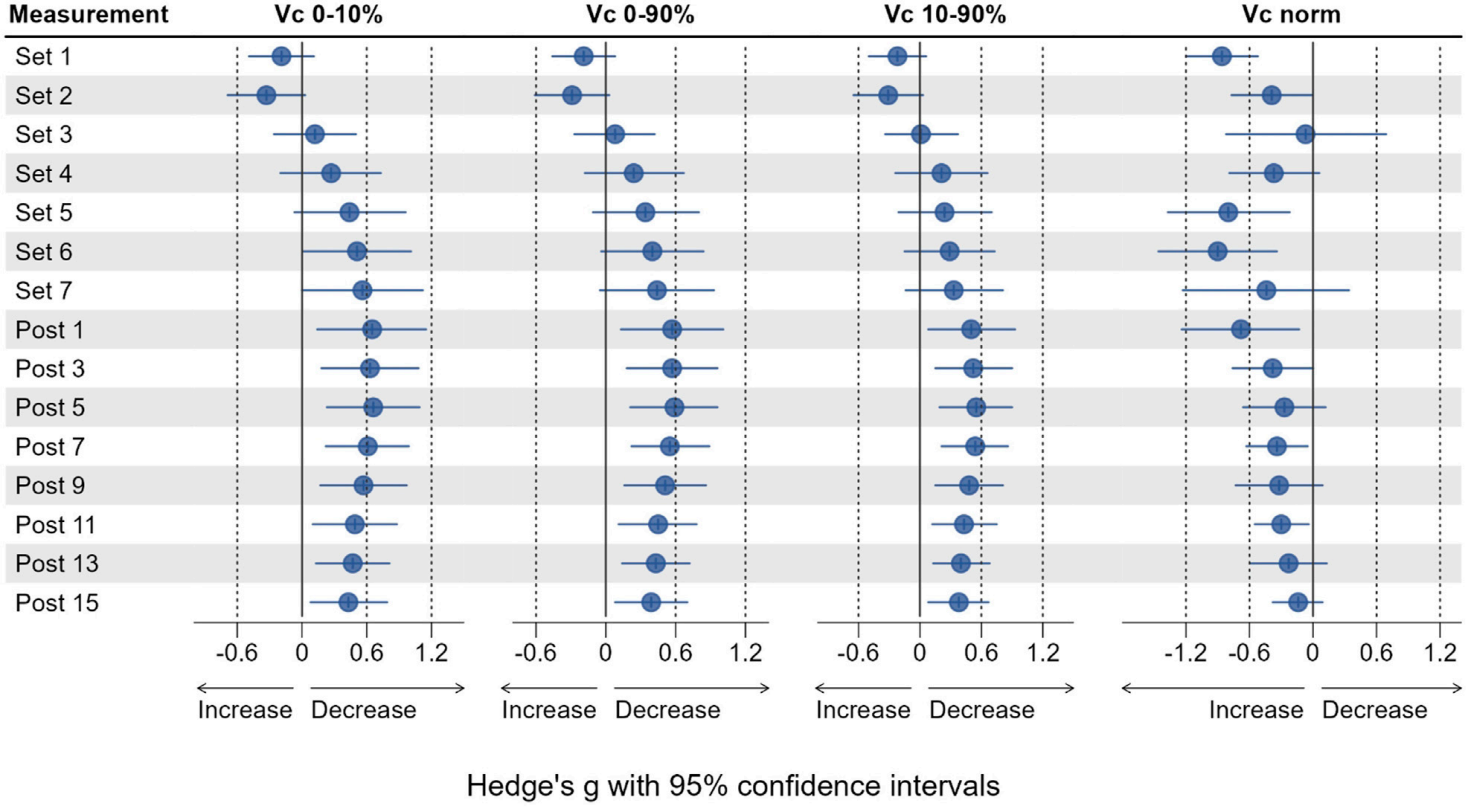
Effect sizes for Vc concepts, representing the change from baseline (Pre 2). Dots represent the estimates of the effect size, horizontal lines represent the 95% confidence intervals.

Vc_0–10%_ did not significantly change from Pre two at any time point (see Figure 5), but was increased by 16.5% at Set 1% and 34.3% Set 2. Vc_0–10%_ then decreased from Set 3 to Post 15 with respective effect sizes ranging from trivial to moderate (Figure 6).

Vc_0–90%_ followed the same pattern with effect sizes ranging from trivial to moderate but was significantly decreased by 36.4% at Post 5 (*p* = 0.05) and 23.3% at Post 7 (*p* = 0.039). Vc_10–90%_ was increased by 21.7% at Set 1% and 34.7% Set 2 (both *p* > 0.05) compared to baseline. Vc_10–90%_ was decreased from Set 3 to Post 15, reaching statistical significance only at Post 7 (*p* = 0.038) with a reduction of 17.6% compared to Pre 2. Effect sizes for changes in Vc_10–90%_ ranged from trivial to moderate in both directions (see Figure 6).

### 3.3 Correlation

**FIGURE 7:**
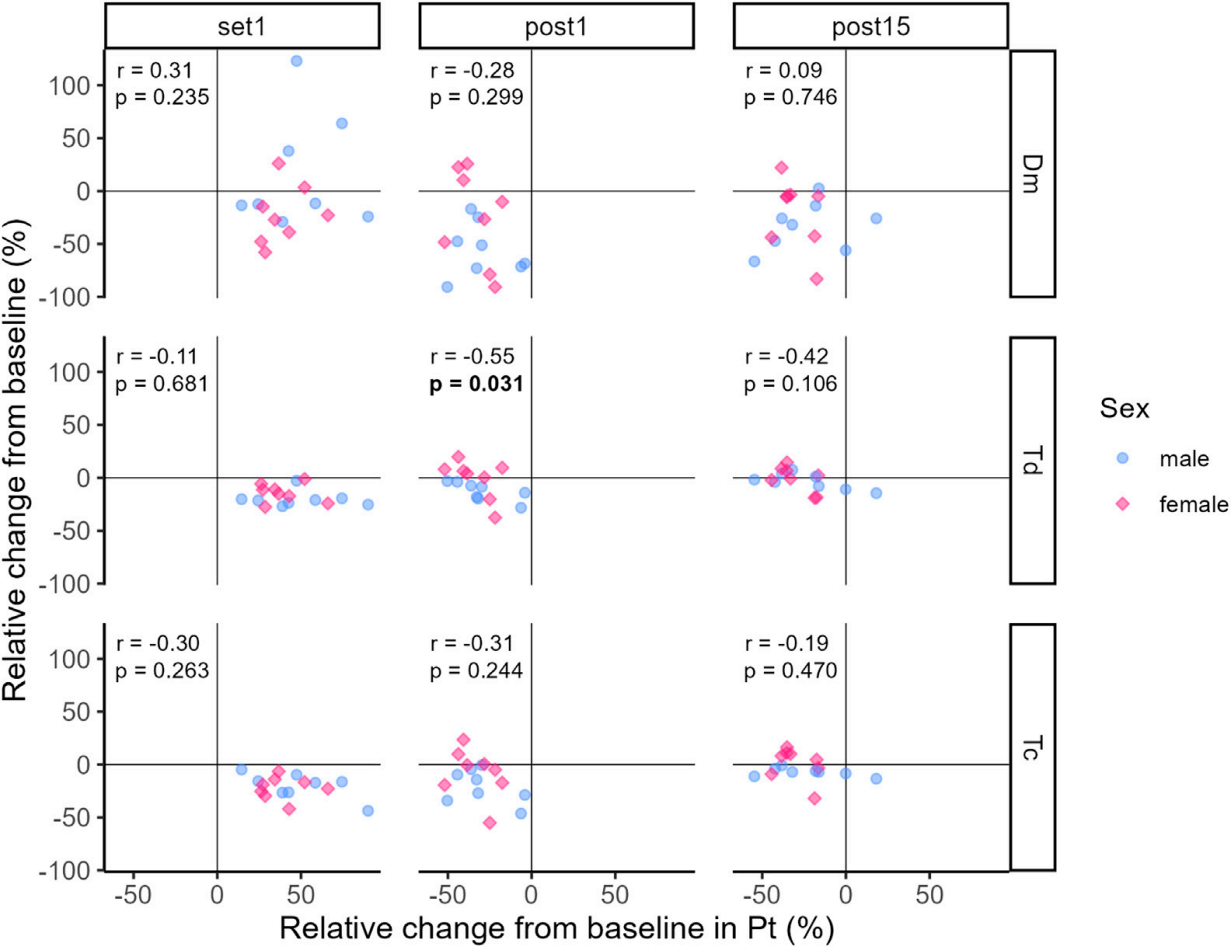
Correlation between relative changes from baseline in Pt and Dm, Td and Tc, respectively. Dots and diamonds represent individual relative changes. The correlation analysis was not grouped by sex, but individual data points are shaped and coloured by sex following the SAGER guidelines.

**FIGURE 8:**
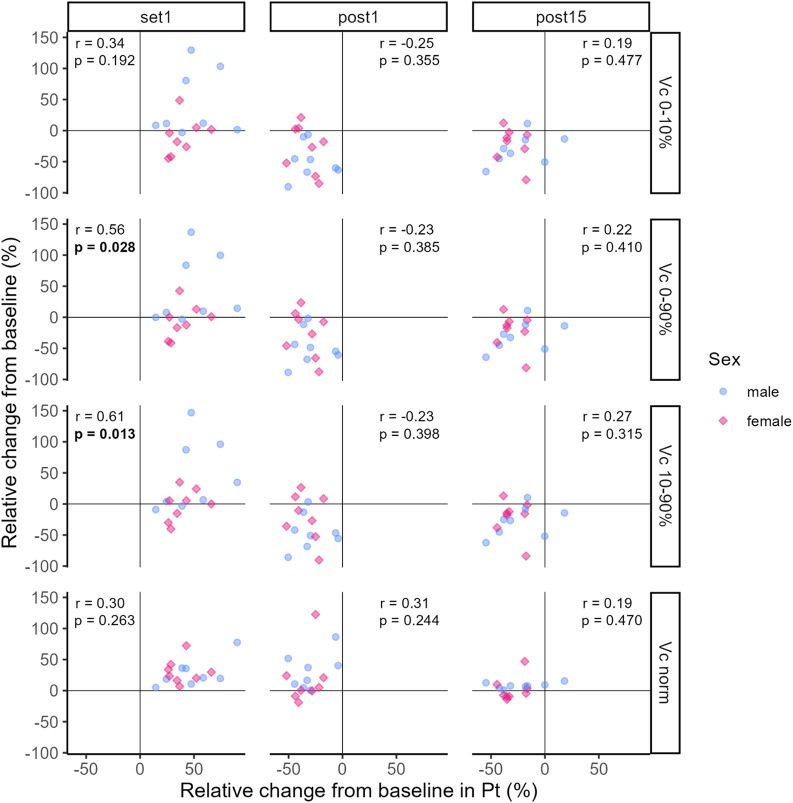
Correlation between relative changes from baseline in Pt and Vc_0–10%_, Vc_0–90%_, Vc_10–90%_, Vc_norm_ respectively. Dots and diamonds represent individual relative changes. The correlation analysis was not grouped by sex, but individual data points are shaped and coloured by sex following the SAGER guidelines.

The correlation coefficients representing the relationship between relative changes from baseline in Pt and TMG-parameters at Set 1, Post 1 and Post 15 are illustrated in Figures 7, 8.

At Set 1, only relative changes in Vc_0–90%_ and Vc_10–90%_ showed significant positive moderate and strong relationships to relative changes in Pt, respectively (see Figure 8). That is, an increase in Pt above baseline was accompanied by increases in Vc_0–90%_ and Vc_10–90%_. For the remaining TMG-parameters, relationships were trivial to moderate and not significant at Set 1 (see Figures 7, 8). At Post 1, a significant moderate negative relationship between relative changes in Td and Pt was found, i.e., the decrease in Pt below baseline was associated with a decrease in Td. At the same time point, the relationships between relative changes in the other TMG-parameters and Pt were mostly moderate negative but not significant. At Post 15, relative changes in TMG-Parameters showed trivial to moderate positive (Dm and Vc variables) or negative (Td and Tc) relationships to changes in Pt with none of them reaching statistical significance.

### 3.4 Diagnostic accuracy

The markers of diagnostic accuracy for twitch displacement parameters at Set 1, Post 1 and Post 15 are presented in Table 5. At Set 1, based on Pt (i.e., the reference test), all 16 participants were classified as potentiated. Consequently, only S_n_ and DE could be calculated as markers of diagnostic accuracy. Tc and Vc_norm_ showed the highest DE across TMG parameters, as all 16 participants were correctly classified as potentiated according to these two parameters. DE of the remaining TMG parameters ranged from 0.19 for Td to 0.94 for Vc_0–10%_ and Vc_10–90%_. At Post 1, 14 of 16 participants were classified as fatigued based on changes in Pt. According to Tc and Vc_norm_, 15 and 16 of 16 participants were falsely classified as not fatigued, respectively. Consequently, DE was the lowest for these two parameters. Across all TMG parameters, only Vc_10–90%_ was able to correctly classify more than 50% of the participants as fatigued (DE = 0.56) at Post 1. The AUROC for Vc_10–90%_ at Post 1 was 0.54 and thus represented bad diagnostic accuracy. At Post 15, according to Pt, 14 of 16 participants were still classified as fatigued. DE at Post 15 was 0.50 or lower, meaning that none of the TMG parameters correctly classified more than 50% of the participants as fatigued.

**TABLE 5:** Diagnostic accuracy markers for twitch displacement parameters at Set 1, Post 1 and Post 15.

Time point	Variable	S_n_ (95% CI)	S_p_ (95% CI)	PPV (95% CI)	NPV (95% CI)	DE (95% CI)	YI (95% CI)	AUROC
Set 1	Dm	0.81 (0.54–0.96)	-	-	-	0.81 (0.54–0.96)	-	-
	Td	0.19 (0.04–0.46)	-	-	-	0.19 (0.04–0.46)	-	-
	Tc	1.00 (0.79–1.00)	-	-	-	1.00 (0.79–1.00)	-	-
	Vc_0–10%_	0.94 (0.70–1.00)	-	-	-	0.94 (0.70–1.00)	-	-
	Vc_0–90%_	0.88 (0.62–0.98)	-	-	-	0.88 (0.62–0.98)	-	-
	Vc_10–90%_	0.94 (0.70–1.00)	-	-	-	0.94 (0.70–1.00)	-	-
	Vc_norm_	1.00 (0.79–1.00)	-	-	-	1.00 (0.79–1.00)	-	-
Post 1	Dm	0.36 (0.13–0.65)	0.50 (0.01–0.99)	0.83 (0.36–1.00)	0.10 (0.00–0.45)	0.38 (0.15–0.65)	−0.14 (−0.86 to 0.64)	0.57
	Td	0.29 (0.08–0.58)	0.00 (0.00–0.84)	0.67 (0.22–0.96)	0.00 (0.00–0.31)	0.25 (0.07–0.52)	−0.71 (−0.92 to 0.42)	0.86
	Tc	0.07 (0.00–0.34)	1.00 (0.16–1.00)	1.00 (0.03–1.00)	0.13 (0.02–0.40)	0.19 (0.04–0.46)	0.07 (−0.84 to 0.34)	0.54
	Vc_0–10%_	0.43 (0.18–0.71)	0.00 (0.00–0.84)	0.75 (0.35–0.97)	0.00 (0.00–0.37)	0.38 (0.15–0.65)	−0.57 (−0.82 to 0.55)	0.79
	Vc_0–90%_	0.57 (0.29–0.82)	0.00 (0.00–0.84)	0.80 (0.44–0.97)	0.00 (0.00–0.46)	0.50 (0.25–0.75)	−0.43 (−0.71 to 0.67)	0.29
	Vc_10–90%_	0.57 (0.29–0.82)	0.50 (0.01–0.99)	0.89 (0.52–1.00)	0.14 (0.00–0.58)	0.56 (0.30–0.80)	0.07 (−0.70–0.81)	0.54
	Vc_norm_	0.00 (0.00–0.23)	1.00 (0.16–1.00)	-	0.12 (0.02–0.38)	0.12 (0.02–0.38)	0.00 (−0.84 to 0.23)	0.50
Post 15	Dm	0.36 (0.13–0.65)	1.00 (0.16–1.00)	1.00 (0.48–1.00)	0.18 (0.02–0.52)	0.44 (0.20–0.70)	0.36 (−0.71 to 0.65)	0.68
	Td	0.14 (0.02–0.43)	0.00 (0.00–0.84)	0.50 (0.07–0.93)	0.00 (0.00–0.26)	0.12 (0.02–0.38)	−0.86 (−0.98 to 0.27)	0.93
	Tc	0.00 (0.00–0.23)	1.00 (0.16–1.00)	-	0.12 (0.02–0.38)	0.12 (0.02–0.38)	0.00 (−0.84 to 0.23)	0.50
	Vc_0–10%_	0.43 (0.18–0.71)	1.00 (0.16–1.00)	1.00 (0.54–1.00)	0.20 (0.03–0.56)	0.50 (0.25–0.75)	0.43 (−0.67–0.71)	0.71
	Vc_0–90%_	0.43 (0.18–0.71)	0.50 (0.01–0.99)	0.86 (0.42–1.00)	0.11 (0.00–0.48)	0.44 (0.20–0.70)	−0.07 (−0.81 to 0.70)	0.54
	Vc_10–90%_	0.29 (0.08–0.58)	0.50 (0.01–0.99)	0.80 (0.28–0.99)	0.09 (0.00–0.41)	0.31 (0.11–0.59)	−0.21 (−0.90 to 0.57)	0.61
	Vc_norm_	0.00 (0.00–0.23)	1.00 (0.16–1.00)	-	0.12 (0.02–0.38)	0.12 (0.02–0.38)	0.00 (−0.84 to 0.23)	0.50

S_n_: Sensitivity; S_p_: Specificity; PPP: Positive predictive value; NPV: Negative predictive value; DE: Diagnostic effectiveness; YI: Youden’s index; AUROC: Area under the receiver operating characteristics curve.

## 4 Discussion

This study aimed to compare how the RF’s muscle displacement determined via TMG and the simultaneous torque output are affected by the interaction of potentiation and fatigue. We hypothesized that repeated MVICs of the knee extensors would initially induce potentiation, reflected by increased twitch amplitudes, which would be compensated by fatigue as the exercise progressed, reflected by decreased twitch amplitudes. We also hypothesized that TMG parameters derived from the twitch displacement curve could accurately confirm the presence or absence of a potentiation or fatigue condition as determined by Pt.

Our results showed that the exercise protocol initially induced potentiation of the RF, as reflected by an increase in Pt above baseline. As the exercise progressed, Pt gradually decreased to below baseline levels, reflecting a gradual shift from potentiation to fatigue. In turn, TMG parameters derived from the muscle displacement during the same twitch responses were considerably less affected by the exercise. Consequently, TMG parameters could not accurately detect the presence of a potentiated or fatigued condition of the RF compared to the reference marker Pt. We suggest that the low diagnostic accuracy of TMG parameters in our study might be explained by a reduced signal-to-noise ratio at a knee flexion of 90° and associated long muscle length.

In our study, we adopted the exercise protocols used in two previous studies (Siracusa et al., 2019; Abazovic et al., 2022) to ensure that the protocol would initially induce potentiation of the RF, followed by a shift towards fatigue as the exercise progresses. In the study by Abazovic et al. (2022), one set of five 5 s MVICs produced an increase in the stimulated VM and VL Pt of 63%–74.4% above baseline. In two other studies, 60 repeated 5 s MVICs reduced the maximum voluntary and electrically stimulated knee extensor torque to 36.2% ± 8.7% and 42.5% ± 11.5% (Chalchat et al., 2020) and 38.4% ± 12.6% and 49.4% ± 12.6% (Siracusa et al., 2019) below baseline levels, respectively. Our results confirm that the protocol, although slightly adjusted, effectively potentiated the RF after the first set of MVICs, as Pt was increased to 44.1% above baseline. Further, as MVIC torque and Pt were reduced to 39.8% and 31.5% at the end of the exercise and Post 1, our results confirm that the protocol effectively induced a shift towards fatigue. Pt even remained reduced to 26.4% below baseline at 15 min after the exercise had ended. The slightly smaller changes in Pt in our study compared to the studies mentioned above may be explained by the fact that our sample included both men and women. The magnitude of exercise-induced potentiation and fatigue effects on the twitch Pt have been shown to be related to the fibre distribution, with higher proportions of type II fibres being associated with greater potentiation and fatigue (Hamada et al., 2003). A recent meta-analysis reported that, compared to men, women present a higher proportion of type I muscle fibres and a lower proportion of type II muscle fibres (Nuzzo, 2024). Therefore, the comparatively smaller effects of potentiation and fatigue on Pt in our study may be explained by the inclusion of women in our sample. Apart from sex-related factors, the participants’ strength training experience may also have influenced our results. Evidence from a systematic review with meta-analysis suggests that the effect of PAP is greater in stronger individuals with more resistance training experience compared to weaker and less experienced individuals (Seitz and Haff, 2016). It has been further suggested that the increased susceptibility to PAP and fatigue in stronger and more resistance-trained individuals may be linked to a higher proportion of fast-twitch fibres in these individuals compared to untrained individuals (Tillin and Bishop, 2009). As our participants were mainly classified as intermediate or advanced in terms of their strength training experience, the effects of PAP and fatigue on the twitch Pt may have been smaller in untrained individuals. However, conclusive evidence on the long-term effect of resistance training on fibre-type transition is lacking (Plotkin et al., 2021; Folland et al., 2008).

Further, while changes in Pt confirmed the presence of potentiation and fatigue, TMG parameters were considerably less affected by the exercise in our study. These results contrast the reports of Abazovic et al. (2022) and Kalc et al. (2023), showing that TMG parameters could detect potentiation of the VM and VL and fatigue of the VL, respectively, as confirmed by the stimulated Pt in these studies. In contrast to these previous studies, we investigated the RF because it is one of the most frequently studied muscles in TMG research on the reliability and measurement error of TMG (Martín-Rodríguez et al., 2017), the diagnostic accuracy, validity, and reliability of TMG for fatigue assessment (Lohr et al., 2019), and the rate of muscle displacement assessed using TMG (Langen et al., 2022). In selecting this muscle, we intended to favour the relevance of our findings for researchers and practitioners using TMG. Concerning the discrepancies between our results and the findings of Abazovic et al. (2022) and Kalc et al. (2023), we suggest they may be explainable by differences in the test setup, specifically regarding the knee angle and the associated muscle length during the twitch measurements. Both previous studies used a smaller knee angle of 40° (Abazovic et al., 2022) and 60° (Kalc et al., 2023) knee flexion, i.e., a more extended knee, compared to our setup (90° of knee flexion). While knee angles ranged from 30° to 100° of flexion within studies using TMG to determine the knee extensor rate of displacement, 60° of knee flexion was the most employed knee angle within those studies (Langen et al., 2022). The smaller knee angle in previous studies would have resulted in a shorter length of the knee extensor muscles than in our study. Previous studies have shown that the joint angle, i.e., the associated muscle length, affects TMG parameters (Latella et al., 2019; Ditroilo et al., 2011). Generally, the amplitude of Dm was reduced at longer muscle lengths compared to shorter muscle lengths (Latella et al., 2019; Ditroilo et al., 2011). In line with these reports, the greater knee flexion in our setup resulted in a lower amplitude of Dm, i.e. 3.06 ± 1.86 mm and 2.88 ± 1.73 mm at Pre 1 and Pre 2, respectively, compared to reports of Dm amplitudes of the RF in previous studies (Martín-San Agustín et al., 2020; Herring et al., 2021; García-García et al., 2018; Paula Simola R.Á. et al., 2015; Martín-San Agustín et al., 2018; Wilson et al., 2019b). For example, Martín-San Agustín et al. (2020) reported a Dm of 10.26 ± 1.42 mm for the RF of recreational athletes, measured in a supine position at 60° of knee flexion. In another study including recreational athletes, Dm of the RF was 11.67 ± 1.74 mm in women and 9.82 ± 2.01 mm in men, measured in a supine position at 60° knee flexion (Martín-San Agustín et al., 2018). In a study including resistance-trained men, the RF’s Dm was 10.1 ± 3.0 mm, measured in a supine position at 60° knee flexion (Muñoz-López et al., 2021). Interestingly, Dm of the RF was 6.1 ± 1.5 mm, 6.6 ± 1.6 mm and 7.0 ± 2.4 mm in non-resistance trained recreational athletes, measured in a supine position at 60° knee flexion (Wilson et al., 2019b).

Further, muscle length has also been shown to affect potentiation and fatigue (Place et al., 2005; Rassier, 2000; Pethick et al., 2021). While the effect of potentiation on knee extensor Pt appears to be greater at shorter muscle lengths (Place et al., 2005; Rassier, 2000), muscle fatigue has been shown to have a greater impact on Pt at longer muscle lengths of the knee extensors (Pethick et al., 2021). As we aimed to induce both potentiation and fatigue in our study, a smaller knee angle, i.e., a more extended knee, may have been more effective in detecting potentiation but at the same time less effective in terms of fatigue and *vice versa*.

Considering the smaller effect of the exercise on TMG parameters compared to Pt in our study, we suggest that the lower amplitude of Dm has negatively affected the signal-to-noise ratio of TMG parameters. Consequently, the ability of the TMG parameters to detect exercise-induced changes was reduced, which has negatively impacted their diagnostic accuracy in our study.

However, in the absence of a standardized measurement protocol in the literature, our results highlight an important methodological consideration regarding the setup for TMG measurements. The joint angle, i.e., the associated muscle length, apparently affects TMG parameters’ ability to detect changes in contractile properties of the RF due to potentiation and fatigue. So far only a few studies have investigated the effect of different joint angles, i.e., muscle lengths, on TMG parameters (Ditroilo et al., 2011) or reproducibility of TMG parameters (Latella et al., 2019). However, to our knowledge, the effect of joint angle, i.e., muscle length, on TMG parameters’ ability to detect changes in contractile properties has not been investigated. Therefore, we suggest that further research is needed to explore the effect of different joint angles on TMG parameters’ ability to detect changes in skeletal muscles’ contractile properties and their diagnostic accuracy reference to other markers of muscle function.

Further, while the diagnostic accuracy of TMG parameters in our study was insufficient, our results are in line with previous studies investigating the diagnostic accuracy of TMG-derived variables (Kalc et al., 2023; Wiewelhove et al., 2017; Raeder et al., 2016; Wiewelhove et al., 2015). In these studies, the diagnostic efficacy, i.e., the proportion of correctly classified participants among all participants (Šimundić, 2009), of Dm, Td, Tc, Vc_0–10%_ and Vc_0–90%_ ranged from 0.36 to 0.74, 0.66 to 0.76, 0.4 to 0.68, 0.36 to 0.71, 0.36 to 0.71, respectively. In our study, DE was 0.44 and 0.38 for Dm, 0.12 and 0.25 for Td, 0.12 and 0.19 for Tc, 0.50 and 0.38 for Vc_0–10%_, 0.50 and 0.44 for Vc_0–90%_ at Post1 and Post15, respectively. When comparing these results, it is noteworthy that the previous studies on the diagnostic accuracy of TMG variables to detect muscle fatigue have used different reference markers. That is, Wiewelhove et al. (2015) used the repeated sprint ability, i.e., the mean peak velocity of six 4 s sprints, Raeder et al. (2016) used the estimated one repetition maximum in a back squat, Wiewelhove et al. (2017) used the maximum countermovement jump height and Kalc et al. (2023) used the peak torque output during an electrically stimulated double twitch of the knee extensors as reference marker to discriminate fatigued and non-fatigued participants.

Considering the respective focus of the studies by Wiewelhove et al. (2015), Wiewelhove et al. (2017) and Raeder et al. (2016), adopting a context-specific marker to detect fatigue is reasonable. Nevertheless, as previously suggested by Lohr et al. (2019), some of the reference measures previously used to assess the diagnostic accuracy of TMG parameters may represent a confounding factor that may have added to their overall insufficient accuracy. That is, the extent and persistence of muscle fatigue are dependent on the activation or stimulation frequency used during the assessment of muscle function (Skurvydas et al., 2016; Edwards et al., 1977; Westerblad et al., 1993; Ruiter et al., 2005). More specifically, during prolonged low-frequency force depression, the decrease in force produced at low frequencies is more pronounced and longer lasting than at higher frequencies (Edwards et al., 1977). Consequently, when comparing TMG to a criterion measure that involves voluntary maximal contractions, the difference in the activation/stimulation frequencies may contribute to the lack of agreement when discriminating fatigued from non-fatigued subjects.

Further, while previous studies investigated the diagnostic accuracy of TMG parameters in terms of fatigue, our study is the first to investigate their diagnostic accuracy regarding post-activation potentiation. Therefore, we suggest that there is a need for future studies focusing on the diagnostic accuracy of TMG parameters to detect changes in contractile properties not limited to muscle fatigue. Also, we suggest that studies focused on the diagnostic accuracy of TMG-derived parameters should adopt a reference marker specific to the construct assessed by TMG, i.e., contractile properties of single muscle belly.

## 5 Limitations

This study has several limitations. Firstly, while we included more participants than needed as per the *a priori* sample size calculation, a larger sample size would have improved the precision of the estimation of statistical markers. Also, a larger sample size would have allowed a better assessment of diagnostic accuracy by providing a more realistic representation of the prevalence of the conditions studied. Secondly, the electrical stimulation intensity corresponded to the individual intensity required to achieve maximum muscle displacement or the maximum output of the TMG stimulator. Accordingly, the maximum twitch torque may not have been obtained in all cases. In addition, the stimulator’s maximum output was reached in 10 of 16 participants (62.5%) in our study. Thus, the maximum displacement may not have been achieved in these cases either. Third, our results are limited to the rectus femoris muscle and do not necessarily apply to other muscles due to anatomical differences.

## 6 Conclusion

To our best knowledge, this is the first study to investigate how muscle displacement assessed via TMG is affected by the interaction of potentiation and fatigue in direct comparison to the torque produced during the same twitch. An exercise protocol of 60 repeated MVICs of the knee extensors effectively induced an initial potentiation of the RF, which shifted toward fatigue throughout the exercise. Potentiation and fatigue were reflected by a significant increase and decrease in Pt compared to baseline levels, respectively. In contrast, the exercise affected TMG parameters considerably less, which could not accurately detect potentiated or fatigued participants. We suggest that the insufficient diagnostic accuracy of the TMG parameters resulted from a reduced signal-to-noise ratio due to a knee flexion angle of 90° and the associated long muscle length of the RF during the measurements. These results highlight an important methodological consideration, as the ability of TMG parameters to detect changes in contractile properties appears to be influenced by the joint angle, i.e., the length of the respective muscle. Future studies should further investigate the effect of the joint angle, i.e., muscle length, on TMG parameters’ ability to detect changes in contractile properties on their diagnostic accuracy with respect to other markers of muscle function.

## Data Availability

The datasets presented in this study and the code used to perform the data analyses are openly accessible at the following link: https://doi.org/10.17605/OSF.IO/SA8V5.

## Abbreviations

ANOVA, Analysis of variance; AUROC, Area under receiver’s operating characteristics curve; CI, Confidence interval; DE, Diagnostic effectiveness; Dm, First peak of the radial displacement curve; FI, Fatigue index; ICC, Intraclass correlation coefficient; IPAQ-SF, International Physical Activity Questionnaire short form; M, mean; MDC, Minimal detectable change; MVIC, Maximum voluntary isometric contraction; NPV, Negative predictive value; PAP, Post-activation potentiation; PPV, Positive predictive value; Pt, Peak twitch torque; RF, Rectus femoris; SAGER, Sex and gender equity in research; Sn, Sensitivity; Sp, Specificity; SEM, Standard error of measurement; SD, Standard deviation; Tc, Contraction time; Td, Delay time; TMG, Tensiomyography; Vc: Rate of displacement; VM, Vastus medialis; VL, Vastus lateralis; YI, Youden’s index.
